# Transcriptional regulation of bone formation by the osteoblast-specific transcription factor Osx

**DOI:** 10.1186/1749-799X-5-37

**Published:** 2010-06-15

**Authors:** Chi Zhang

**Affiliations:** 1 Bone Research Laboratory, Texas Scottish Rite Hospital for Children, Department of Orthopedic Surgery, University of Texas Southwestern Medical Center at Dallas, Texas, USA

## Abstract

Bone formation is a complex developmental process involving the differentiation of mesenchymal stem cells to osteoblasts. Osteoblast differentiation occurs through a multi-step molecular pathway regulated by different transcription factors and signaling proteins. Osx (also known as Sp7) is the only osteoblast-specific transcriptional factor identified so far which is required for osteoblast differentiation and bone formation. *Osx *knock-out mice lack bone completely and cartilage is normal. This opens a new window to the whole research field of bone formation. Osx inhibits Wnt pathway signaling, a possible mechanism for Osx to inhibit osteoblast proliferation. These reports demonstrate that Osx is the master gene that controls osteoblast lineage commitment and the subsequent osteoblast proliferation and differentiation. This review is to highlight recent progress in understanding the molecular mechanisms of transcriptional regulation of bone formation by Osx.

## Introduction

Bone formation takes place through two distinct processes: endochondral ossification involving a cartilage model and intramembranous ossification by which bones form directly from condensations of mesenchymal cells without a cartilage intermediate. Bone formation is a highly regulated process involving the differentiation of mesenchymal stem cells to osteoblasts. Osteoblasts produce a characteristic extracellular collagenous matrix that subsequently becomes mineralized after hydroxyapatite crystals deposition. Much progress has been made in understanding the factors that control the gene expression program through the osteoblast induction, proliferation, differentiation, and maturation. Osteoblast differentiation occurs through a multistep molecular pathway regulated by different transcription factors and signaling proteins (Table 1). Indian hedgehog (Ihh) is required for endochondral but not for intramembranous bone formation [1] and is needed for the establishment of the osteogenic portion of the perichondrium/periosteum and for the initial activation of the gene for Runx2. Runx2 is needed for the formation of both endochondral and membranous skeletal elements. In *Runx2*-null mutants, no endochondral and no membranous bones form [2]. *Runx2 *is required for the differentiation of mesenchymal cells into preosteoblasts. As a downstream gene of *Runx2*, *Osx *is required for the differentiation of preosteoblasts into mature osteoblasts. *Osx *is specifically expressed in all osteoblasts. In *Osx*-null embryos, cartilage is formed normally, but the embryos completely lack bone formation [3]. Wnt signaling is also essential to osteoblast differentiation during embryonic development. Conditional inactivation of *β-catenin *in either skeletal progenitor cells or at a later stage of osteoblast development in mouse embryos blocks osteoblast differentiation [4-7]. Other transcription factors involved in osteoblast differentiation include Twist1, ATF4, SatB2, Shn3, and Dlx5 [8-12]. This review focuses mainly on the molecular mechanisms of transcriptional regulation of bone formation by Osx.

**Table 1:** Transcription factors and mouse models associated with osteoblast differentiation

Gene	Phenotype on osteoblasts (OB) in knock-out mice	Role	citation
Ihh	reduced chondrocyte proliferation, maturation of chondrocytes at inappropriate position, and failure of OB development in endochondral bones	required for endochondral but not for intramembranous bone formation	1
Runx2	devoid of OB and impaired chondrocyte differentiation	required for OB differentiation of mesenchymal cells into preosteoblasts	2
Osx	completely lack bone formation and cartilage is normal	required for differentiation of preosteoblasts into mature OB	3
β-catenin	block OB differentiation and develop into chondrocyte	important for OB differentiation, and prevent transdifferentiation of OB into chondrocyte	4-7
Twist1	leads to premature OB differentiation	antiosteogenic function by inhibiting Runx2 function during skeletogenesis	8
ATF4	delayed bone formation during embryonic development and low bone mass throughout postnatal life	critical regulator of OB differentiation and function	9
SatB2	both craniofacial abnormalities and defects in OB differentiation and function	a molecular node in a transcriptional network regulating skeletal development and OB differentiation	10
Shn3	adult-onset osteosclerosis with increased bone mass due to augmented OB activity	a central regulator of postnatal bone mass	11

Dlx5	delayed ossification of the roof of the skull and abnormal osteogenesis	positive regulator in OB differentiation	12

## Osx is an osteoblast-specific transcription factor

**Figure1:**
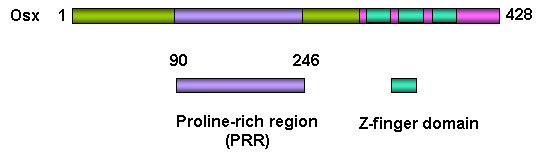
**Domain structure of osteoblast-specific transcription factor Osx**. The DNA-binding domain of Osx is located at its C terminus containing three Z-finger domains and there is a proline-rich region (PRR) close to N terminus in Osx.

*Osx *was discovered as a bone morphogenic protein-2 (BMP2) induced gene in mouse pluripotent mesenchymal cells, encoding a transcription factor that is highly specific to osteoblasts [3]. Osx is also expressed at low level in pre-hypertrophic chondrocytes. The *Osx *gene is located in chromosome 15 in mouse and in chromosome 12 in human. There are only two exons in the *Osx *gene. Exon 1 sequence encodes the seven N-terminal amino acids of Osx, and exon 2 contains the remaining open reading frame (ORF) and 3-prime UTR. The mouse Osx protein is a 428 amino acid polypeptide with a molecular mass of about 46 kDa as shown in Figure1. The DNA-binding domain of Osx is located at its C terminus and contains three C2H2-type zinc finger domains that share a high degree of identity with a similar motif in Sp1, Sp3, and Sp4. There is a proline-rich region (PRR) close to the N-terminus. Osx binds to functional GC-rich sequences similar to the consensus binding sites of erythroid Krüppel-like factor (EKLF) and Sp1. The subcellular localization of Osx is restricted to the nucleus. The PRR region is responsible for the Osx inhibitory effect on the Wnt signaling pathway [13].

During mouse embryogenesis, *Osx *transcripts are not detected before embryonic stage E13 [3]. *Osx *first appears in differentiating chondrocytes, the surrounding perichondrium, and mesenchymal condensations of future membranous bones of E13.5 embryos. After E15.5, *Osx *is strongly expressed in cells that are associated with all bone trabeculae and bone collar formation. Weak expression of *Osx *is observed in the prehypertrophic zone. *Osx *is highly expressed in bone trabeculae and in secondary ossification centers after birth. *Osx *is only expressed in cells in the bone matrix and the inner (endosteum) and outer (periosteum) bone surfaces.

## *Osx *is required for bone formation and osteoblast differentiation

It has been demonstrated that Osx is necessary for bone formation and mineralization in vivo [3]. The *Osx *gene was inactivated in the mouse embryonic stem (ES) cells using homologous recombination to understand Osx function. Most of the exon2 coding sequence was deleted. As a result, the *Osx *gene was inactivated. Heterozygous *Osx *mutant mice were normal and fertile. Homozygous *Osx *mutant mice were lethal and these mice had difficulty in breathing, rapidly became cyanotic, and died within 15 min of birth. Newborn homozygous mutant mice showed severe inward bending of forelimbs and hindlimbs [3]. Although *Osx*-null embryos have normal cartilage development, they completely lack bone formation, so neither endochondral nor intramembranous bone formation occurs. The mesenchymal cells in *Osx*-null mice do not deposit bone matrix, and cells in the periosteum and the condensed mesenchyme of membranous skeletal elements cannot differentiate into osteoblasts. In the endochondral skeletal elements of *Osx*-null mutants, a dense mesenchyme emerges from the perichondrium/periosteum and invades the zone of hypertrophic chondrocytes along with blood vessels. However, cells in this mesenchyme are arrested during differentiation. A similar, dense mesenchyme is also found in the membranous skeletal elements. Bone trabeculae are completely absent in all skeletal elements. Although mineralization does not occur in membranous skeletal elements, it does in the endochondral skeleton because of the physiological mineralization of the zone of hypertrophic chondrocytes. No mineralization occurs in the periosteum, suggesting that bone collars do not form.

In *Osx*-null mutant embryos, expression of type I collagen (*Col1a1*) in the condensed mesenchyme of the membranous skeleton and the periosteum and mesenchyme of the endochondral skeleton is severely reduced. Expressions of the osteoblast-specific markers such as osteonectin, osteopontin and bone sialoprotein (BSP) cannot be detected in these mesenchymes. In E18.5 *Osx*-null embryos, osteocalcin, a late, highly specific osteoblast marker, is not expressed in endochondral and membranous skeletal elements. Despite a lack of osteoblast markers expression, *Runx2 *expression in *Osx*-null mutants remains comparable to that of wild-type osteoblasts in the dense mesenchyme of both membranous and endochondral skeletal elements. Thus, osteoblast differentiation is completely arrested in *Osx*-null embryos, even though similar expression of *Runx2 *remains compared to wild-type embryos. On other hand, over-expressed Osx *in vitro *has been shown to induce expression of osteocalcin and collagen type 1a1.

In the skeletal elements of E18.5 *Osx*-null embryos the number of TRAP-positive cells appear to be reduced compared to wild-type embryos. In long bones of *Osx*-null embryos cells from the periosteum invade the zone of the hypertrophy chondrocyte as a wedge-shaped expansion of the periosteum in which osteoblast precursors are arrested in their differentiation. These observations are supported by the evidence that expressions of both OPG and RANKL are downregulated, but the ratio of OPG/RANKL increases in E18.5 *Osx*-null calvarial cells [13]. Expression of the osteoclast marker TRAP is also downregulated. Thus, it is possible that the inhibition of Wnt signaling by Osx also reduces osteoclast differentiation and function. It is speculated that the inhibition of Wnt signaling by Osx, which itself has an essential role in osteoblast differentiation, insures an optimal bone formation rate.

## Osx inhibits osteoblast proliferation during bone development

It has been demonstrated that canonical Wnt signaling is required for normal osteoblast proliferation. A marked increase in osteoblast proliferation occurs when β-catenin is stabilized in osteoblasts during mouse embryonic development [6]. Moreover *Lrp5-*null mice, which phenocopy the osteoporosis-pseudoglioma syndrome in humans [14], develop a phenotype with low bone mass due to decreased osteoblast proliferation [15]. In contrast, gain-of-function mutants of *Lrp5 *lead to high bone mass syndrome in patients [16] and in mice [17]. The Wnt signaling antagonist Dkk1 prevents the activation of Wnt signaling by binding to LRP5/6. It has been shown that the bone formation and bone mass of heterozygous *Dkk1 *mutant mice increase with an increased number of osteoblasts [18]. In contrast, the overexpression of *Dkk1 *in osteoblasts causes severe osteopenia with decreased osteoblast numbers [19]. These data indicate that Wnt signaling stimulates osteoblast proliferation.

Recent studies in our research group have provided evidences showing that the osteoblast-specific transcription factor Osx is able to inhibit Wnt pathway activity during osteoblast differentiation [13]. In calvarial cells of E18.5 *Osx*-null embryos, expression of the Wnt antagonist *Dkk1 *was abolished, and that of Wnt target genes *c-Myc *and *cyclin D1 *was increased. It has been demonstrated that Osx binds to and activates the *Dkk1 *promoter. Osx is shown to inhibit β-catenin-induced Topflash reporter activity and also inhibit β-catenin-induced secondary axis formation in *Xenopus *embryos. Moreover, this study showed that in calvaria of E18.5 *Osx*-null embryos harboring the *TOPGAL *reporter transgene, β-galactosidase activity was increased, suggesting that Osx inhibited the Wnt pathway in osteoblasts *in vivo *[13]. Osx can disrupt Tcf binding to DNA, providing a likely mechanism for the inhibition by Osx of β-catenin transcriptional activity. The transcription factor Tcf is known to interact with β-catenin to form a functional complex in promoter region of Wnt signaling targets to activate gene expression.

**Figure2:**
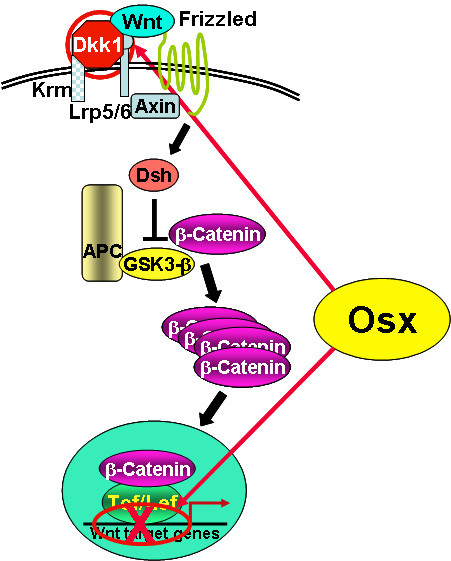
**Model of mechanisms of the Osx inhibitory effect on Wnt pathway**. Osx negatively controls Wnt pathway by two different mechanisms: activates the expression of Wnt antagonist Dkk1 and disrupts Tcf binding to DNA to inhibit the transcriptional activity of β-catenin/Tcf.

The PRR region of Osx is responsible for disruption of Tcf1 binding to DNA, and for inhibition of β-catenin transcriptional activity. These findings indicate that Osx negatively controls the activity of β-catenin in two different mechanisms shown in Figure2: first, by being needed for the expression of a major Wnt antagonist and second, by inhibiting the transcriptional activity of β-catenin/Tcf.

We have shown that Osx decreases osteoblast proliferation [13]. E18.5 *Osx*-null calvaria showed greater BrdU incorporation than wild-type calvaria, and primary calvarial cells from *Osx*-null E18.5 embryos also grew faster than wild-type cells. On the other hand, Osx over-expression in C2C12 mesenchymal cells inhibited cell growth. Because Wnt signaling has a major role in stimulating osteoblast proliferation, it is speculated that Osx-mediated inhibition of osteoblast proliferation is a consequence of the Osx-mediated control of Wnt/β-catenin activity. These results add a new layer of control to Wnt signaling in bone formation.

## Molecular pathway of osteoblast differentiation

Osx is necessary for the osteoblast lineage [3,13]. Following the lineage commitment, osteoprogenitors undergo a proliferative stage. Subsequently, they exit mitosis, transit to express genes such as alkaline phosphatase (*ALP*), bone sialoprotein (*BSP*) and type I collagen, as they commence to produce and mature osteogenic extracellular matrix. Finally, they express genes involved in mineralization of the extracellular matrix such as osteocalcin (*OC*), osteopontin. This highly regulated program of gene expression and cellular differentiation is governed by the expression and activity of different transcription factors. These factors do not act alone but interact with each other to integrate diverse signals and fine-tune gene expression.

**Figure3:**
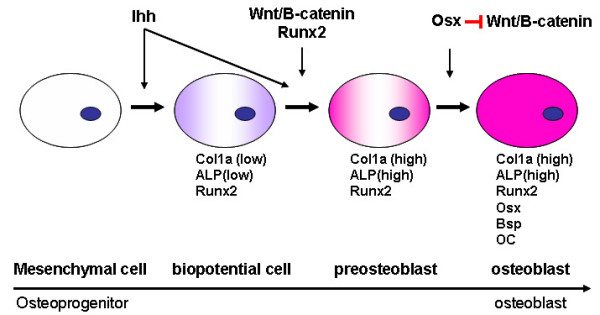
**The proposed model of coordinated regulation of osteoblast differentiation and proliferation during bone formation by Osx and Wnt/β-catenin signaling**. Ihh is the initiator of endochondral ossification. The *Runx2*-expressing biopotential progenitors can differentiate into either osteoblast or chondrocyte. Then cells differentiate into preosteoblasts, in which Runx2 play an essential role. In the next step, preosteoblasts differentiate into mature osteoblast, a process in which Osx plays a critical role. Wnt/β-catenin signaling has an essential role in osteoblast differentiation and osteoblast proliferation. The inhibition of Wnt/β-catenin signaling activity by Osx constitutes a possible mechanism for the inhibition by Osx of osteoblast proliferation.

Based on the characterization of the *Osx*-null mutant phenotype and recent studies, the following brief model for osteoblast differentiation is proposed as shown in Figure3. Ihh is the initiator of endochondral ossification. Osteoblast progenitors in mesenchymal condensations differentiate first into biopotential progenitors in which Runx2 starts to express. These *Runx2*-expressing biopotential progenitors can differentiate into either osteoblast or chondrocyte depending on cell signaling. Then cells differentiate into preosteoblasts, a process in which Runx2 play an essential role. At this stage, preosteoblasts express early osteoblast marker genes like *ALP*. Next step, preosteoblasts differentiate into mature osteoblast, a process in which Osx plays a critical role. Mature functioning osteoblasts strongly express characteristic later osteoblast marker genes such as *OC *and *BSP*. In the membranous and endochondral skeletons, *Osx*-null preosteoblasts are blocked from differentiating into osteoblasts, so there is no mature osteoblast without *Osx*. In *Osx*-null embryos, osteoblast differentiation markers, such as OC, BSP and osteonectin, are not expressed. Because the promoter regions of several osteoblast marker genes contain binding sites for Runx2 that are functional in DNA transfection experiments [20,21], it is possible that the Runx2 and other transcription factors, act with Osx to activate osteoblast marker genes *in vivo *and produce a bone-specific matrix.

Wnt/β-catenin signaling has an essential role in osteoblast differentiation during embryonic development and has a major role in stimulating osteoblast proliferation during both embryonic and postnatal development. Osx is an osteoblast-specific transcription factor, required for osteoblast differentiation. The inhibition of Wnt/β-catenin signaling activity by Osx, also constitutes a possible mechanism for the inhibition of osteoblast proliferation by Osx. Recent observations that Osx inhibits Wnt signaling pathway in vitro and in vivo provide novel concept of feedback control mechanisms involved in bone formation [13].

Osx is believed to be downstream of Runx2 in the pathway of osteoblast differentiation because Runx2 expression is normal in *Osx*-null mice, while no *Osx *transcripts are detected in skeletal elements in *Runx2*-knockout mice [3]. This is confirmed through characterization of a Runx2-binding element in the *Osx *gene promoter [22]. It is not known yet which transcription factors are downstream target of Osx.

## Regulation of Osx expression in osteoblasts

The mechanism underlying the regulation of Osx expression in osteoblasts is still unclear. Several studies have reported that some factors can modulate Osx expression. Both BMP-2 and insulin-like growth factor-1 (IGF-1) can induce Osx expression in undifferentiated mesenchymal stem cells [23]. IGF-I-mediated Osx expression required all three MAPK components (Erk, p38, and JNK), whereas BMP-2 required p38 and JNK signaling. Blocking Runx2 activity inhibited the BMP-2-mediated induction of Osx, suggesting a Runx2-dependent pathway. However, another research group showed that BMP-2 induced Osx expression through a Runx2-independent pathway [24]. Even if Osx has been suggested as a downstream target of Runx2, the results of this study indicated that Osx expression was still induced by BMP-2 treatment in Runx2 null cells but not induced by Runx2 over-expression in C2C12 cells. Regulatory mechanisms of BMP-2 on Osx are not yet fully understood. Ascorbic acid and 1,25(OH)_2 _vitamin D_3_, which have positive roles in osteoblast function, have also been shown to up-regulate Osx expression [25,26]. It was demonstrated that Ascorbic acid induced Osx expression via a novel mechanism involving Nrf1 nuclear translocation and Nrf1 binding to an antioxidant-responsive element to activate genes critical for cell differentiation.

Some studies indicate that negative regulators of osteoblastogenesis can inhibit Osx expression. TNF inhibited Osx mRNA in pre-osteoblastic cells without affecting Osx mRNA half-life [27,28]. Inhibitors of MEK1 and ERK1, but not of JNK or p38 kinase, abrogated TNF inhibition of Osx mRNA and promoter activity. *In vivo *studies provide genetic evidence that p53 tumor suppressor blocks osteoblast differentiation and bone development [27,28]. Prolonged exposure to parathyroid hormone (PTH) negatively regulates Osx expression in osteoblasts by a transcriptional mechanism mediated by cAMP signaling [29]. PTH inhibited Osx mRNA and protein expression, and this effect could be mimicked by forskolin, 8-bromo-cAMP, or expression of constitutively active Gsalpha. On the other hand, some other researchers found that systemic PTH treatments accelerated fracture healing in mice concomitantly with increased Osx expression in the PTH treated fracture calluses, suggesting a mechanism for PTH-mediated fracture healing possibly via Osx induction [30]. Recently studies indicated that intermittent PTH increased in vivo Osx expression in osteoblasts through a pathway requiring activating transcription factor 4 (ATF4) [31]. ATF4-responsive element has been identified in the proximal *Osx *promoter.

Despite these interesting findings, the details concerning the regulation and function of Osx are incompletely understood.

## Osteoporosis and Osx

Osteoporosis is characterized by reduced bone mass, alterations in the microarchitecture of bone tissue, reduced bone strength, and an increased risk of fracture [32]. Osteoporosis is a common condition that affects up to 30% of women and 12% of men at some point in life. The prevalence of osteoporosis increases with age due to an imbalance in the rate at which bone is removed and replaced during the bone remodeling, which is an important physiological process essential for healthy skeleton maintenance. Many factors influence the risk of osteoporosis--including diet, physical activity, medication use, and coexisting diseases--but one of the most important clinical risk factors is a positive family history, emphasizing the importance of genetics in the pathogenesis of osteoporosis. Genetic factors have been recognized to play important roles in the pathogenesis of osteoporosis. Evidence from twin and family studies suggests that between 50% and 85% of the variance in peak bone mass is genetically determined [33].

Recent study has indicated that genetic variants in the chromosomal region of *Osx *are associated with bone mineral density (BMD) in children and adults probably through primary effects on growth [34]. A genome-wide association study of BMD and related traits in 1518 children from the Avon Longitudinal Study of Parents and Children (ALSPAC) was carried out to identify genetic variants affecting BMD. This research group identified associations with BMD in an area of chromosome 12 containing the *Osx *(*SP7*) locus. A meta-analysis of these existing studies revealed strong association between SNPs in the *Osx *region and adult lumbar spine BMD. In light of these findings, this research group genotyped a further 3692 individuals from ALSPAC who had whole body BMD and confirmed the association in children as well.

Although Osx has been identified to be associated with osteoporosis-related phenotypes, further investigation needs to be done to determine whether Osx will represent a useful diagnostic index of osteoporosis or molecular target for therapeutic manipulation.

## Possible clinical application of Osx

Osx is indispensable for the commitment of the osteoblast lineage and the expression of the osteoblast-specific matrix proteins, including type I collagen, bone sialoprotein, osteonectin, and osteocalcin. No pharmacological approach to target Osx in osteoblasts has been reported. Heterozygous mutations in *Runx2 *, which is an upstream of *Osx*, have been shown to be the cause of the human genetic disease cleidocranial dysplasia [35]. There is no evidence so far that any *Osx *mutation leads to any clinical human disease.

The extensive studies by many laboratories to explore how to control the Wnt signaling pathway in osteoblasts stems from the realization that this pathway has an essential role in bone mass determination in the adult skeleton. There is also an expectation that efforts to pharmacologically target this pathway should yield promising agents to treat bone diseases such as osteoporosis. Results in our group showing that Osx inhibits Wnt/β-catenin signaling add an important new layer of control to the complex regulation of the Wnt pathway in osteoblasts [13].

It was observed that the Osx expression was decreased in two mouse osteosarcoma cell lines and in three human osteosarcoma cell lines [36]. Transfection of the *Osx *gene into the mouse osteosarcoma cells inhibited tumor cell growth in vitro and in vivo and significantly reduced tumor incidence, tumor volume, and lung metastasis following intratibial injection. Using an in vitro migration assay, Osx suppressed the migration of tumor cells to lung extracts. These results suggest that Osx expression may play a role in osteosarcoma tumor growth and metastasis, and that osteolytic activity of tumor cells may be regulated by Osx via down-regulation of interleukin-1 gene transcription [36]. It is relatively consistent with the recent mechanism studies that Osx inhibits osteoblast proliferation through controlling the Wnt pathway [13].

Bone formation is essential for maintenance and healing of the skeleton following injury and operative interventions, such as osteotomies and limb lengthening. In numerous orthopedic conditions, such as congenital pseudoarthrosis of tibia, femoral head osteonecrosis, and large bone lengthening, bone healing and regeneration remain challenging goal to achieve. Most therapy for skeletal diseases with less bone such as osteoporosis and osteonecrosis is aimed at inhibiting bone resorption, but to cure these diseases, it is also critically important to stimulate new bone formation. Therefore, there is currently great interest in understanding the regulation of osteoblast differentiation and activity to guide the development of anabolic therapies. Although no pharmacological approach to target Osx in osteoblasts has identified yet, an interesting future research direction is to look for upstream genes or molecules which can selectively target Osx expression and activity. We speculate that Osx could become a therapeutic target in efforts to stimulate the anabolic pathway of bone synthesis.

## Conclusions

Bone formation is a complex process regulated by multiple factors and pathways; it is clearly shown that Osx is required for the final commitment of osteoblast lineage. Although recent molecular and genetic studies using gene targeting in mice have established Osx as a master regulator of osteoblast differentiation during bone formation, the mechanisms of Osx regulation of osteoblast differentiation and function are still under investigation. Future studies to decipher the Osx direct upstream or downstream molecular targets, Osx expression regulation and Osx functional partners are required to clarify the detailed mechanism of the temporal and spatial regulation of Osx for bone formation and homeostatic regulation of skeletal system. The need to develop novel drugs that stimulate bone formation and thereby elevate bone mass (anabolic agents) has opened new research areas for therapeutic intervention in the treatment of bone-related diseases.

## Abbreviations

Osx: Osterix; OB: osteoblast; E18.5: embryonic day 18.5; Ihh: Indian hedgehog; *Col1a1: *type I collagen; OC: osteocalcin; BSP: bone sialoprotein; ALP: alkaline phosphatase; PRR: proline-rich region; BMP2: bone morphogenic protein-2; BMD: bone mineral density.

## Competing interests

The authors declare that they have no competing interests.

## Authors' contributions

The author contributed to the article. The author has read and approved the final manuscript.
